# Fungal feeding preferences and molecular gut content analysis of two abundant oribatid mite species (Acari: Oribatida) under the canopy of *Prosopis laevigata* (Fabaceae) in a semi-arid land

**DOI:** 10.1007/s10493-023-00790-7

**Published:** 2023-04-18

**Authors:** Daniel Isaac Sánchez-Chávez, Salvador Rodríguez-Zaragoza, Patricia Velez, Nathalie Cabirol, Margarita Ojeda

**Affiliations:** 1 Laboratorio de Ecología Microbiana, Unidad de Biotecnología y Prototipos, FES Iztacala, UNAM, 54090 Mexico City, Mexico; 2 Departamento de Botánica, Instituto de Biología, UNAM, 04510 Mexico City, Mexico; 3 Grupo de Ecología Microbiana Funcional del Suelo y Protección Ambiental, Facultad de Ciencias, UNAM, 04510 Mexico City, Mexico; 4 Laboratorio de Ecología y Sistemática de Microartrópodos, Facultad de Ciencias, UNAM, 04510 Mexico City, Mexico

**Keywords:** Edaphic microbiota, Mite-fungi trophic interaction, Resource partitioning, Soil mites

## Abstract

*Prosopis laevigata* (mesquite; Fabaceae) forms fertility islands in soils of semi-arid lands where microbial diversity concentrates in response to the accumulation of resources in the soil beneath individual plants, promoting organic matter decomposition and nutrient cycling. This phenomenon provides suitable conditions for the proliferation of key edaphic elements such as fungi and mites. Mite-fungal interactions are central for our understanding of nutrient cycling processes in resource-limited arid food webs; yet, no information is available about fertility islands in semi-arid lands. Thus, we aimed to determine in vitro fungal-based feeding preferences and molecular gut content of the oribatid mite species *Zygoribatula* cf. *floridana* and *Scheloribates* cf. *laevigatus*, which are abundant under the canopy of *P. laevigata* in an intertropical semi-arid zone in Central Mexico. Our results on the gut content analysis of these oribatid species resulted in the ITS-based identification of the following fungi: *Aspergillus homomorphus*, *Beauveria bassiana*, *Filobasidium* sp., *Mortierella* sp., *Roussoella* sp., *Saccharomyces cerevisiae*, Sclerotiniaceae sp. and *Triparticalcar* sp. Furthermore, under laboratory conditions both oribatid mite species exhibited feeding preferences on melanized fungi, such as *Cladosporium* spp., whereas *A. homomorphus* and *Fusarium penzigi* were avoided. Our findings indicated that the analyzed oribatid mite species have similar feeding preferences for melanized fungi, which might suggest resource partitioning and a degree of preference, explaining the coexistence of both oribatid species.

**Supplementary Information:**

The online version contains supplementary material available at 10.1007/s10493-023-00790-7.

## Introduction

Desert soils are highly susceptible to degradation due to the reduced biological productivity, demanding an efficient nutrient mobilization in a short period of time (Whitford and Duval 2019). In these stressed environments, the canopy and the rhizosphere of trees enhance higher microbial and plant activities under them (fertility island effect), sustaining complex soil food webs during dryness, in contrast to sun-exposed or degraded areas (Ochoa-Hueso et al. 2018). Under these circumstances, complex biotic interactions proliferate in response to environmental stress or degradation pressures.

Plant health and nutrition depend on microbial communities, recycling nutrients via organic matter degradation (Klironomos and Kendrick 1995). In addition, the invertebrate community (mesofauna) facilitates microbial recycling of lignin, tannins, phenols, waxy cuticles, and other polymers (Stott and Martin 1989; Bailey et al. 2002; Schneider et al. 2004a, b; Walter and Proctor 2013). Among this invertebrate community, oribatid mites are acknowledged as important nutrient mobilizers (Hartenstein 1962; Siepel and Maaskamp 1994; Maraun et al. 1998; Schneider et al. 2004a, b).

Oribatid mites are the most abundant and active microarthropods in fertility islands under the arid-active trees *Prosopis laevigata* (Humb. & Bonpl. ex Willd.) M.C. Johnst. (mesquite) even during the driest part of the year in a semi-arid zone from southern Mexico (Rodríguez-Zaragoza et al. unpubl. data). Their activity has a strong impact on the biological productivity in arid systems (Santos and Whitford 1981; Cepeda-Pizarro and Whitford 1989; Klironomos and Kendrick 1995; Todria et al. 2021) where fungal-based food webs are characteristic due to fungal metabolic ability to exploit high molecular weight compounds such as litter and woody structures (Wardle et al. 2004). Oribatid mites, by grazing on fungal hyphae and spores, mobilize nutrients, control fungal dispersion and community structure (Behan-Pelletier and Hill 1978; Renker et al. 2005; Vašutová et al. 2019). Hence, oribatid mites enhance the decomposition rates, nutrient cycling, microbial respiration, and plant growth (Siepel and Maaskamp 1994; Zak and Whitford 1988; Crowther et al. 2012).

*Zygoribatula floridana* and *Scheloribates laevigatus* are cosmopolitan oribatid mite species (Murvanidze et al. 2018; Subias 2022). These taxa have been reported from resource-limited and stressful systems, such as arid and semi-arid areas (Neher et al. 2009; Krantz and Walter 2009) feeding potentially on litter (Hubert et al. 2000; Wickings and Grandy 2011; Gergócs and Hufnagel 2016), lichen, algae (Erdmann et al. 2007), and nematodes (Rockett and Woodring 1966; Muraoka and Ishibashi 1976; Rockett 1980; Ramakrishnan and Neravathu 2019). However, several reports suggest they prefer fungal hyphae and spores over the rest of potential items (Behan-Pelletier and Hill 1978; Siepel and de Ruiter-Dijkman 1993; Hubert et al. 2001, 2004; Maraun et al. 2023), placing them on the fungal-based food web. As fungal consumers, these oribatid mite species might compete for similar resources, posing the possibility of resource partitioning through selective feeding to avoid strong competition (Schneider and Maraun 2005).

Direct in situ observations have been attempted to elucidate mite-fungal interactions and their impact on the soil system (Smrž 1991, 2002; Hubert et al. 2001). However, these approaches face several methodological limitations such as: (1) the cryptic nature of both oribatid mite species in field, and (2) difficulties for distinguishing between parasitic fungi and those serving as food, since fungal and mite morphological identification of both fungi and mite taxa is difficult, time consuming, and the analysis of mites’ gut content is extremely challenging to perform in the field (Remén et al. 2010). At present, these limitations may be overcome by in vitro feeding experiments coupled with the analysis of gut contents through amplification of fungal ITS region (Velez et al. 2018). Moreover, knowledge of mites' fungal diet would provide more insights on the ‘fungal path’ of soil nutrients in semi-arid environments. However, the potential fungal resources on which abundant oribatid taxa feed in the surroundings of *P. laevigata* in a semiarid land remain unknown. Hence, we aimed to determine fungal taxa associated with oribatid mites *Z.* cf. *floridana* and *S.* cf. *laevigatus*, collected under the canopy of *P. laevigata* in a preserved and a degraded terrace during rainy season from an intertropical semiarid soil, as well as to determine their feeding preferences on the isolated fungi under laboratory conditions. We hypothesized that each of the terraces harbors a characteristic acarofauna that establishes close trophic interactions with the local mycobiota. This work provides major insights into how the oribatid mite species coexist within their specific assemblage.

## Materials and methods

**Fig. 1:**
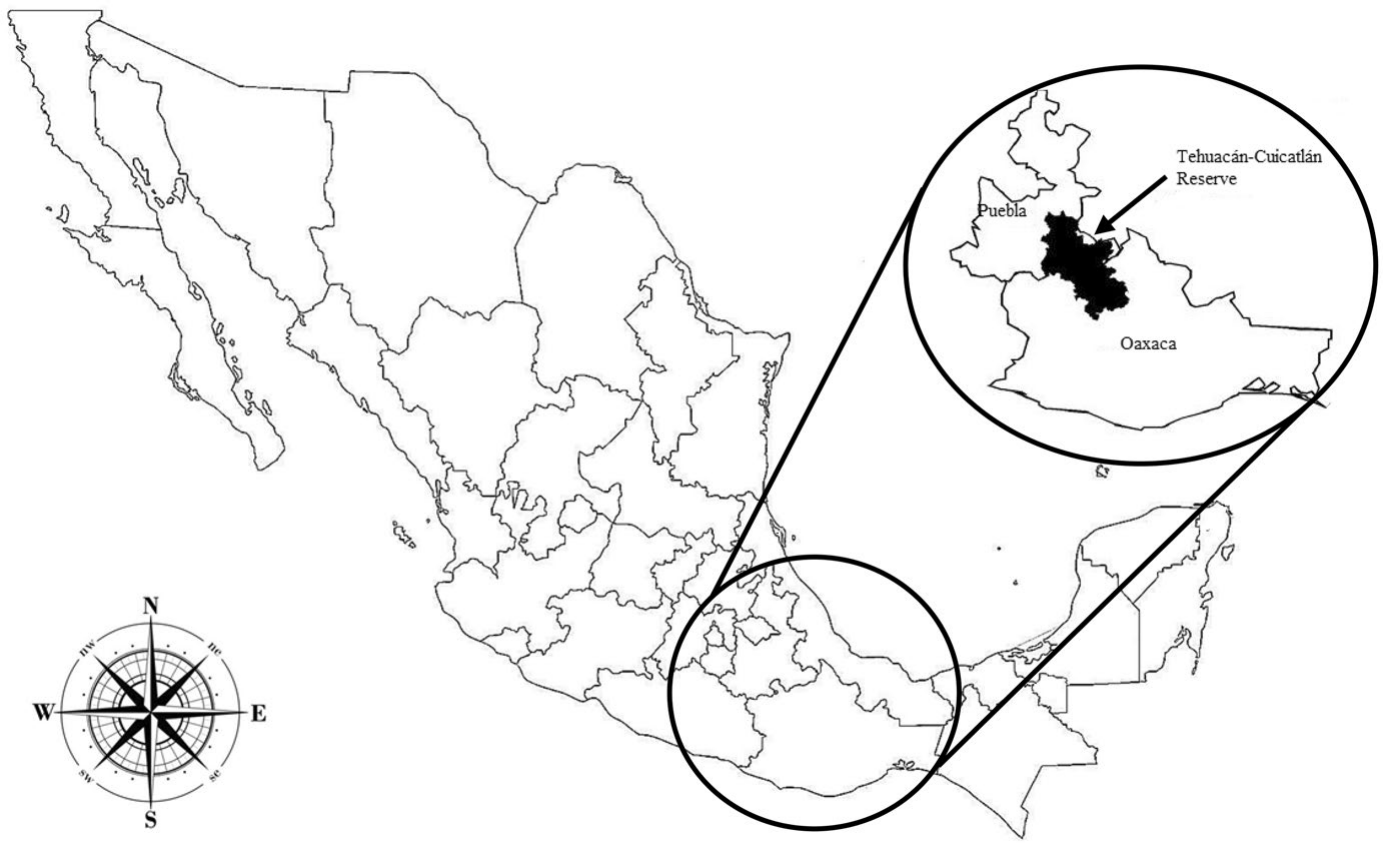
Map of Tehuacán-Cuicatlán Biosphere Reserve located south of the State of Puebla, Mexico (18.326064, −97.453120)

Zapotitlán belongs to the Tehuacán-Cuicatlán Valley Reserve, located in the southeast of the State of Puebla and northeast of the State of Oaxaca, Mexico (Fig. 1). Semi-aridity of this zone is due to the rain shadow effect, producing a 4-month rainy season each year. Climate is dry-hot with summer rains, corresponding to Köppen’s Bs class (Peel et al. 2007). The valley has an average temperature of 21 °C and 400–450 mm average rainfall (García 2004). Vegetation is xerophytic shrubland, dominated by legume trees like mesquite (*P. laevigata*), Palo Verde (*Parkinsonia praecox*), *Mimosa luisiana* and several species of cacti (Dávila et al. 2002).

The Zapotitlán area displays alluvial terraces deposited by ‘El Salado’ river during the Pleistocene (López-Galindo et al. 2003) with patches of well-developed herbaceous, shrub and scattered tree species (conserved patches) intermixed with patches having scarce or no vegetation (degraded patches). Several of these patches are transitioning into badlands (López-Galindo et al. 2003), where the fertility island effect of *P. laevigata* is well observed (González-Ruiz et al. 2008).

Samplings were conducted during the season of heavy rains (late May-late July 2018). Individual litter-soil samples were collected under the canopy of six *P. laevigata* trees at 10 cm depth from the conserved terrace (18°19′30.0″N, 97°27′12.3″W) and six more, with the same conditions, from the degraded one (18°19′34.8″N, 97°27′17.1″W). Samples were deposited in 2-kg Ziploc bags and transferred to the Laboratory of Microbial Ecology, FES Iztacala, UNAM, for extraction and sorting of oribatid mites.

Microarthropods were extracted from the samples using the Berlese funnel extraction method for 4 days and collected into plastic vials. Half of the vials contained 90% alcohol for mite preservation and identification (Crossley and Blair 1991). The other half of the vials contained moistened filter paper to collect mites alive for culturing and feeding preferences tests.

For morphological identification, adult specimens were, first, macerated with lactic acid for increasing transparency and a better microscopic observation. Second, mites were transferred to permanent slide mounts with Hoyer’s medium for their identification (Krantz and Walter 2009). The keys of Franklin et al. (2008) and Balogh and Balogh (1990) were used to identify the specimens by morphology achieving identification to genus or species level.

From the mites collected in vials with alcohol, 12 mites from each of the two most abundant taxa were separated and individually stored in 2-ml Eppendorf tubes with 100% alcohol, for gut dissection and molecular analysis. Cuticles of these mites were sanitized prior to gut dissection to ensure the absence of external contamination for DNA analysis of gut content. Thus, individual mites were transferred to 2-ml Eppendorf tubes and vortexed 30 s in a solution of 1 ml sterile water and 0.01% Tween 80, for a period of 30 s twice in 1 ml sterile water (Velez et al. 2018). Subsequently, under sterile conditions and using a stereomicroscope (Nikon), the exoskeleton was opened by inserting an insect needle (no. 00) in the base of the notogaster (separating the ventral and dorsal plates of the body), revealing the gut. Next, the gut was extracted with another sterile insect needle and placed individually in Eppendorf tubes for DNA extraction.

Additionally, another 12 specimens corresponding to the most abundant oribatid taxa were extracted and their cuticles were processed for fungal isolation, due to its potential to carry fungi (Remén et al. 2010). Each individual mite was placed in a sterile Eppendorf tube containing a solution of 1 ml distilled water and 0.01% Tween 80, and then vortexed for 1 min. Subsequently, 500 µl was plated into Petri dishes with Potato Dextrose Agar (PDA; supplemented with 0.5 g/ml of benzathine benzylpenicillin to prevent bacterial growth) and incubated at room temperature (22 ± 3 °C) for 2 weeks. Developing fungi were isolated and grouped based on the macroscopic characteristics. We selected and sub-cultured the top-20 most abundant isolates for subsequent feeding preference tests. All of the isolates were identified and deposited in the Laboratory C-121, Institute of Biology, UNAM, and are available for research upon request. For identification, the genomic DNA of each isolate was obtained using the protocol described by Doyle and Doyle (1987). The ITS1-5.8S-ITS4 region of rDNA was amplified using the primer set ITS 1 and ITS 4 (5′-TCCGTAGGTGAACCTGCGG-3′ and 5′-TCCTCCGCTTATTGATATGC-3′) (White 1990). PCR products were sequenced in both directions by the Laboratory of Biodiversity and Health Genomic Sequencing, Institute of Biology, UNAM.

The fungal ITS region of DNA mixture obtained from the dissected guts was directly amplified using the Thermo Scientific Phire Animal Tissue kit following the manufacturers’ instructions, with the primer set ITS 1 and ITS 4 (White et al. 1990). In this experiment, we included positive (*Cladosporium cladosporioides*) and negative (no mite’s gut in the tube mix) controls. The PCR products were visualized on a 1% agarose gel stained with GelRed to verify amplification products. Electrophoresis revealed amplification in nearly all samples, but only strongly stained and well-defined bands of amplified DNA were considered for sequencing in the Laboratory of Biodiversity and Health Genomic Sequencing.

Fungal sequences from both experiments (fungal isolation and gut content analysis) were manually edited and trimmed using BioEdit software (v.7.2; Hall 1999), and were compared to the GenBank database, using Basic Local Alignment Search Tool (BLASTn) (Altschul et al. 1990). For each OTU, several sequences, preferably from published studies, with a minimum cut-off value of 98–100% for presumed species and 94–97% for genus level, were considered (Millberg et al. 2015).

**Fig. 2:**
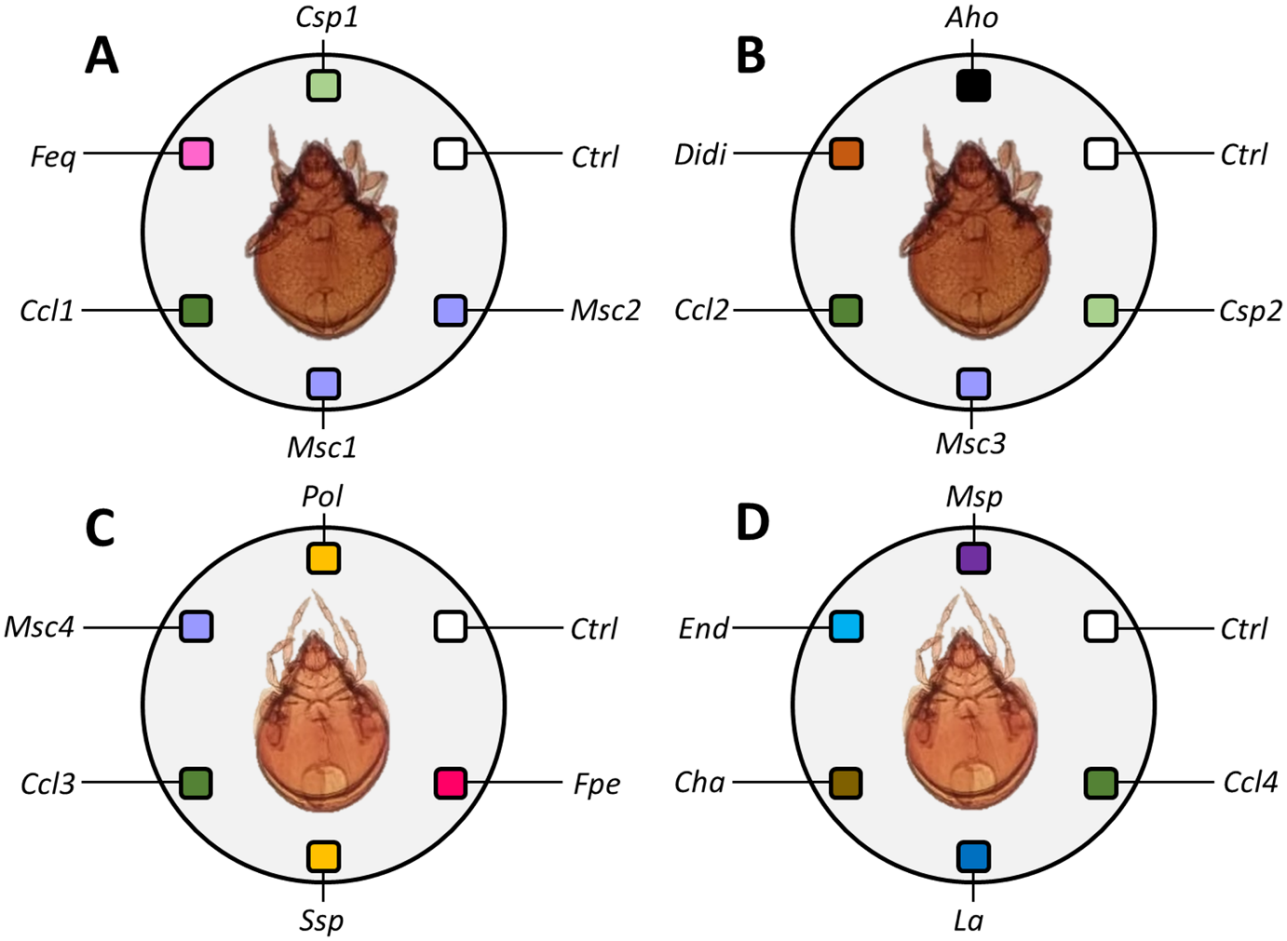
Feeding preference tests including five fungal species and a control (baker’s yeast). *Zygoribatula* cf. *floridana* collected in a conserved area (**A**) and a degraded area (**B**). *Scheloribates* cf. *laevigatus* collected in a conserved area (**C**) and in a degraded area (**D**). *Aspergillus homomorphus* (Aho), Chaetomiaceae sp. (Cha), *Cladosporium cladosporioides* (Ccl), *Cladosporium* sp. (Csp), control (Ctrl), Didmellaceae sp. (Didi), Endomycetaceae sp. (End), *Fusarium equisetum* (Feq), *Fusarium penzigii* (Fpe), *Leptosphaerulina australis* (La), *Mortierella schmuckeri* (Msc), *Mortierella* sp. (Msp), *Penicillium olsonii* (Pol), *Sarcopodium* sp. (Ssp)

Adult specimens of the abundant taxa collected in vials with moistened filter paper were sorted and subsequently cultured in 100-ml glass vials with Paris plaster and charcoal (9:1), soil litter from the sampling site, and Baker’s yeast as diet. Temperature and humidity in cultures were kept at 20 ± 1 °C and 60 ± 10%, before feeding preference tests (Krantz and Walter 2009). Prior to tests, the oribatid mite specimens in culture were starved for 5 days. Tests were conducted in 100-ml glass containers, where 15 individuals of *Z.* cf. *floridana* and* S.* cf. *laevigatus* mites were added in separated containers. Thus, four tests were conducted according to the sampling site where fungi and oribatid mites were extracted: (A) individuals of *Z.* cf. *floridana* collected from a conserved area, (B) individuals of *Z.* cf. *floridana* from a degraded area, (C) individuals of *S.* cf. *laevigatus* from a conserved area, and (D) individuals of *S.* cf. *laevigatus* collected from a degraded area (Fig. 2). Five replicates were used per test. Each test contained five fungal species collected from their respective oribatid cuticle and baker’s yeast as control (Maraun et al. 1998; Hubert et al. 2001; Schneider et al. 2004a, b). Observations under a stereomicroscope (Nikon) were made every 30 min, until nearly all food items were depleted, so the test duration depended on the intensity of consumption. Furthermore, three criteria were considered to determine feeding preferences: (1) preferred, where > 6 mites in the glass vial stayed consuming the food choice; (2) transitory, where < 6 mites consumed the food choice; and (3) avoided, no mites approached or consumed the food choice.

For this study, sampling sites were not considered variables which might have influenced the feeding preference test, as mites and fungi were cultured afterwards under laboratory conditions and fed with the same diet before tests. Hence, the sampling site effect was disregarded. To approach diversity differences in terms of acarofauna, a principal component analysis (PCA) was performed using R software (R core team 2021) with the *vegan* package (v.2.6–4) (Oksanen et al. 2017) using the oribatid taxa abundance against both terraces (conserved terrace and degraded terrace). Furthermore, a Kruskal–Wallis test was performed to determine significant differences in feeding preferences. Subsequently, a Dunn post-hoc test was used to contrast differences among treatments. Statistical analysis was carried out using R (R Core Team 2021).

## Results

The oribatid mites *Z*. cf. *floridana* and *S*. cf. *laevigatus* were the most abundant oribatid taxa collected under the canopies of *P. laevigata* from both terraces. These species belong to families Oribatulidae and Scheloribatidae, respectively, both with a worldwide distribution (Subias 2022), and accounting for 97 (*Z.* cf. *floridana*) and 75 (*S.* cf. *laevigatus*) individuals (Table S1), being *Z.* cf. *floridana* the most abundant in both sites.

**Fig. 3:**
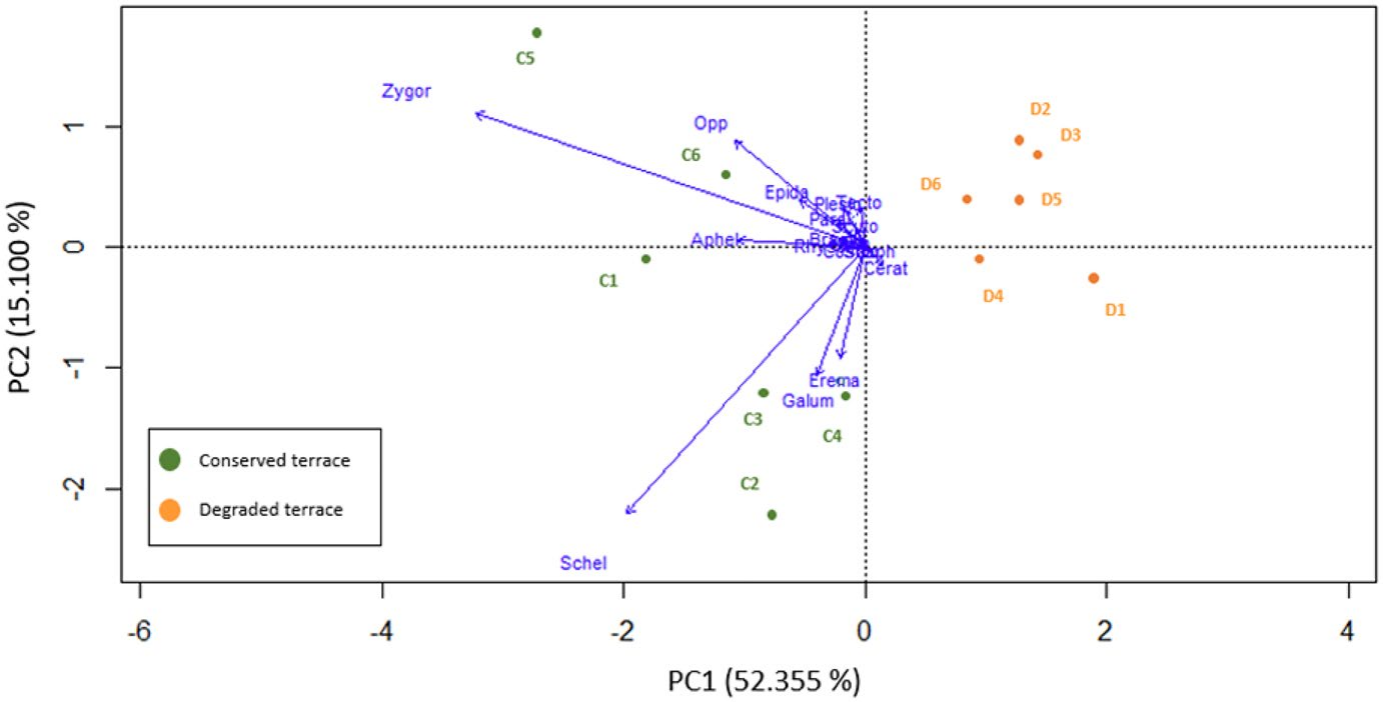
Principal coordinates analysis (PCA) ordination biplot of oribatid mite taxa under the canopy of *Prosopis laevigata* on both terraces. Oribatid mite taxa codes are reported in Table S1 (n = 6)

The PCA biplot (Fig. 3) displayed the differences in oribatid mite taxa assemblage between both terraces. PC1 and PC2 explained 52.4 and 15.1% of the variance, respectively. Nearly half (12 specimens) of the oribatid individuals dissected displayed traces of fungal DNA from gut content DNA analysis. However, only a fraction of DNA amplified from mite gut extractions yielded to fungal DNA suitable material for sequencing and identification, which indicates that fungal DNA might degrade between sample collection and amplification, reducing its detectability. This might also be due to individuals feeding at different times and rates on a variety of sources as they are heterogeneously distributed in soil (Rohde 1959; Anderson and Healey 1972).

The amplified ITS sequences from mites’ gut produced fragments of different sizes, ranging from 500 bp (*Mortierella* sp.) to 200 bp (*Aspergillus* sp.). Based on these sequences, eight fungal species from the gut of oribatid mites were identified: *Beauveria bassiana*, *Filobasidium* sp., *Saccharomyces cerevisiae* and *Triparticalcar* sp. in the gut of *Z.* cf. *floridana*, and *Aspergillus homomorphus*, *Mortierella* sp., *Roussoella* sp. and Sclerotiniaceae sp. in the gut of *S.* cf. *laevigatus* (Table 1).

**Table 1:** Operational taxonomic units** (**OTUs) of fungi extracted from the gut of *Zygoribatula* cf. *floridana* and *Scheloribates* cf. *laevigatus* collected in the field

Phylum	Class	Family	OTU at the species level	NCBI access no.
Ascomycota	Eurotiomycetes	Aspergillaceae	*Aspergillus homomorphus*	MT764764
	Sordariomycetes	Cordycipitaceae	*Beauveria bassiana*	MT764765
	Dothideomycetes	Thyridariaceae	*Roussoella* sp.	MT764763
	Saccharomycetes	Saccharomycetaceae	*Saccharomyces cerevisiae*	MT764766
	Leotiomycetes	Sclerotiniaceae	Sclerotiniaceae sp.	MT764769
Basidiomycota	Tremellomycetes	Filobasidiaceae	*Filobasidium* sp.	MT764767
Mucoromycota	Mortierellomycetes	Mortierellaceae	*Mortierella* sp.	MT764768
Chytridiomycota	Chytridiomycetes	Spizellomycetaceae	*Triparticalcar* sp.	MT764770

Among the 20 fungi isolated from the cuticle of several individuals of both oribatid mites,* C. cladosporioides* was the most representative fungus species, followed by *Mortierella schmuckeri*. Further isolates obtained from the cuticle of mites included: *A. homomorphus* and two unidentified species of *Cladosporium* (sp. 1 and sp. 2), *Fusarium equiseti*, *Fusarium penzigii*, *Leptosphaerulina australis*, *Mortierella* sp., *Penicillium olsoni* and *Sarcopodium* sp. (Table 2).

**Table 2:** Fungi isolated from the cuticle of *Zygoribatula* cf. *floridana* and *Scheloribates* cf. *laevigatus* collected on two terraces, one conserved and one degraded.

Fungal taxa	*Zygoribatula* cf. *floridana*		*Scheloribates* cf. *laevigatus*	
	Conserved terrace	Degraded terrace	Conserved terrace	Degraded terrace
*Aspergillus homomorphus*	−	+	−	−
Chaetomiaceae sp.	−	−	−	+
*Cladosporium cladosporioides*	+	+	+	+
*Cladosporium* sp. 1	+	−	−	−
*Cladosporium* sp. 2	−	+	−	−
Didimellaceae sp.	−	+	−	−
*Fusarium equiseti*	+	−	−	−
*Fusarium penzigii*	−	−	+	−
Endomycetaceae sp.	−	−	−	+
*Leptosphaerulina australis*	−	−	−	+
*Mortierella schmuckeri*	+	+	+	−
*Mortierella* sp.	−	−	−	+
*Penicillium olsonii*	−	−	+	−
*Sarcopodium* sp.	–	–	+	–

All fungi were cultured in PDA and used as a food option in mite feeding preference tests. ‘ + ’ represents presence and ‘−’ represents absence of fungi

**Fig. 4:**
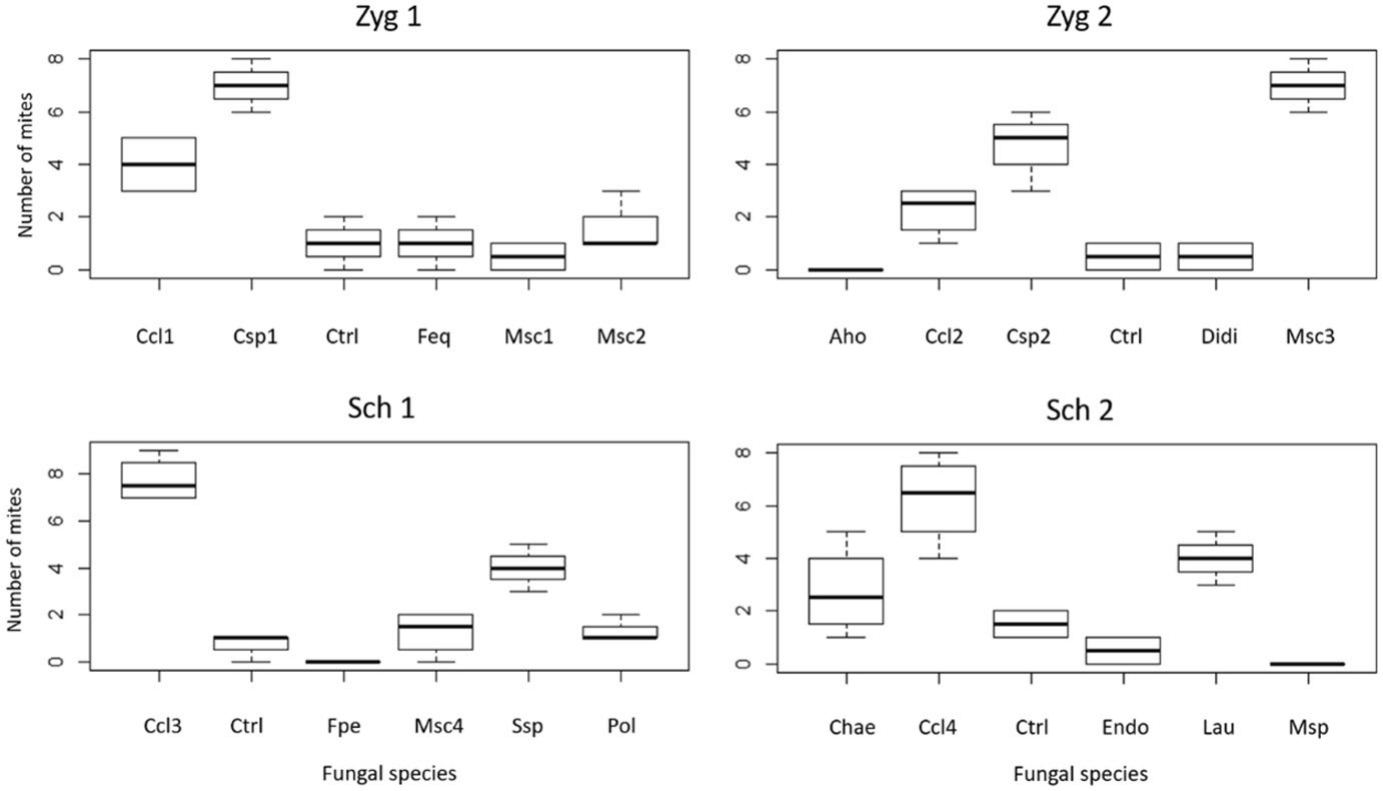
Boxplot depicting feeding preferences of *Zygoribatula* cf. *floridana* (Zyg1 in test 1 and Zyg2 in test 2) and *Scheloribates* cf. *laevigatus* (Sch1 in test 3 and Sch2 in test 4). The boxes display the interquartile range, and the bold line displays the median; whiskers of the boxplots represent the upper and lower quartiles (25th and 75th percentiles). Fungal choices: Aho—*Aspergillus homomorphus*, Chae—Chaetomiaceae sp., Ccl—*Cladosporium cladosporioides*, Csp—*Cladosporium* sp., Ctrl—control, Didi—Didmellaceae sp., Endo—Endomycetaceae sp., Feq—*Fusarium equisetum*, Fpe—*Fusarium penzigii*, Lau—*Leptosphaerulina australis*, Msc—*Mortierella schmuckeri*, Msp—*Mortierella* sp., Pol—*Penicillium olsonii*, Ssp—*Sarcopodium* sp. (n = 4)

Differences in the feeding preferences by the two oribatid taxa were observed (Fig. 4). *Zigoribatula* cf. *floridana* showed a marked preference for *Cladosporium* sp. 1 (test A; H_5, 0.5_ = 25.28, P < 0.05) which was consumed until depletion (Table 3). However, *Z.* cf. *floridana* from test B displayed a strong preference on *M. schmuckeri* (H_5, 0.5_ = 28.78, P < 0.05) whereas *A. homomorphus* was avoided (Table 3).

**Table 3:** Feeding preference of *Zygoribatula* cf. *floridana* in two tests (test A: mites collected from a conserved area; test B: mites collected from a degraded area), both repeated 4 × (P = preferred, T = transitory, A = avoided)

Test	Repetition	*Cladosporium* sp. 1	*Cladosporium cladosporioides*	*Fusarium equisetum*	*Mortierella schmuckeri*	*Mortierella schmuckeri*	Control
A	1	T	P	T	T	T	A
	2	T	T	T	T	A	A
	3	T	P	A	T	T	T
	4	T	P	T	T	A	A

		*Aspergillus homomorphus*	*Cladosporium cladosporioides*	*Cladosporium* sp. 2	Didimellaceae sp.	*Mortierella schmuckeri*	Control
B	1	A	T	T	T	P	T
	2	A	T	T	T	P	A
	3	A	T	T	A	P	T
	4	A	P	T	A	P	A

*Scheloribates* cf. *laevigatus* from test C preferred to feed on *C. cladosporioides* and *Sarcopodium* sp. (H_5, 0.5_ = 26.99, P < 0.05) although neither of these fungi were its first choice. *Scheloribates* cf. *laevigatus* fed from them towards the end of the tests. *Fusarium penzigii* was always avoided in all treatments (Table 4). *Scheloribates* cf. *laevigatus* from test D preferred *C. cladosporioides* and frequently avoided *Mortierella* sp. and *Endomicetacea* sp. (H_5, 0.5_ = 28.21, P < 0.05; Table 4). Baker’s yeast was consumed in nearly all tests, although mites looked first for other suitable food.

**Table 4:** Feeding preference of *Scheloribates* cf. *laevigatus* in two tests (test C: mites collected from a conserved area; test D: mites collected from a degraded area), both repeated 4 × (P = preferred, T = transitory, A = avoided)

Test	Repetition	*Cladosporium cladosporioides*	*Fusarium penzigii*	*Mortierella schmuckeri*	*Penicillium olsonii*	*Sarcopodium* sp.	Control
C	1	T	A	T	A	P	T
	2	P	A	T	T	T	T
	3	P	A	A	T	P	A
	4	P	A	T	T	P	A

		*Cladosporium cladosporioides*	Chaetomiaceae sp.	Endomycetaceae sp.	*Leptosphaerulina australis*	*Mortierella* sp.	Control
D	1	P	T	A	T	A	T
	2	P	T	A	P	A	T
	3	P	T	T	T	T	T
	4	P	T	A	T	A	A

## Discussion

In this study, both *Z.* cf. *floridana* and *S.* cf. *laevigatus* were abundant on both terraces. We hypothesized to find a particular oribatid mites’ community assemblage on both terraces due to different soil degradation processes affecting them. This suggests that *P. laevigata* provides the minimum conditions necessary to harbor a diversity of soil fungi, which is reflected in the abundance of both oribatid taxa on the terraces, since they might feed on a great variety of fungi (Behan-Pelletier and Hill 1978).

Under laboratory conditions, *Z.* cf. *floridana* and *S.* cf. *laevigatus* shared feeding preferences on several fungal species, which might suggest either competition or resource partitioning between these two taxa in their environment. However, resource partitioning is a plausible explanation for their co-occurrence in soil, i.e., high availability and diversity of food resources in a heterogeneous environment, or consumption of fungal spores by one oribatid taxon and consumption of hyphae by the other (Koukol et al. 2009).

Both oribatid mites fed on *C. cladosporioides* until depletion, accompanied by a massive production of feces, which according to Smrž and Norton (2004), might be due to intensive feeding for nutrient assimilation from sources with very low nutrient content which is reflected in a massive production of feces with fragments of partially digested fungal structures. It is likely that *C. cladosporioides* in the field benefits from this mechanism for spore attachment to the mite cuticle while feeding on hyphae, which offers a colonizing advantage for this fungus being dispersed via cuticle transport or fecal deposition, favoring resource colonization and exploitation (Visser et al. 1981; Williams et al. 1998). Preference for melanized taxa such as *Cladosporium* was also reported by Schneider and Maraun (2005).

The fungus *A. homomorphus* was avoided all the time by *Z.* cf. *floridana*, causing mites to seek for other available fungi during the food choices. However, traces of *A. homomorphus* were found in the gut of both oribatid taxa collected in the field*.* Probably, *A*. *homomorphus* faces a trade-off between growing or producing toxic compounds which may repel mites during hyphal development (Koukol et al. 2009; Chatterjee et al. 2016). Thus, this fungus might become a more palatable food to mites if hyphae are produced fast, reducing the quantity and quality of toxic molecules (Behan-Pelletier and Hill 1978; Hubert et al. 2003; Velez et al. 2018).

*Mortierella* spp. hyphae have been reported to be undesirable for microarthropods (Dix and Webster 1995; Maraun et al. 1998; Werner et al. 2016; Yoder et al. 2019). However, in this study *Z.* cf. *floridana* preferred to feed on *Mortierella* sp., and although neither *M. schmuckeri* nor *Mortierella* sp. were their first choice, individual mites fed on them. This degree of preference could explain the occurrence of this fungal species as the second most reported fungal species on the mites’ cuticle.

Few individuals of *Z.* cf. *floridana* fed on* P. olsonii*. However, the interaction among the two taxa is not clear as this fungus has been acknowledged to interact differently with oribatid mites. For example, Hartenstein (1962) and Seniczak et al. (2017) reported that *Penicillium* is repellent to oribatids, whereas Seniczak and Stefaniak (1981) and Hubert et al. (1999) observed that *Penicillium* was attractive to a different set of mite species, which suggests a complex species-specific relationship among the taxa. Thus, further investigations of this isolate are needed to better understand its interaction with soil mites.

The direct amplification of the fungal barcode region from mites’ gut content is a reliable tool to analyze feeding specificity in the mite-fungus interaction. However, certain factors hamper a good DNA identification. i.e., time after the last food intake. Nonetheless, this minor deviation may be corrected by increasing sample size, as well as by feeding preference tests under laboratory conditions. In this sense, our results showed the presence of different fungal species such as *A. homomorphus*, *Mortierella* sp. and *S. cerevisiae*, in the gut of *Z.* cf. *floridana* and *S.* cf. *laevigatus*, which may suggest that the analyzed oribatids fulfill their different nutritional needs based on a mixed-fungal diet that provides an assortment of nutrients to successfully fulfill their life cycle (Gan et al. 2014; Maraun et al. 2020).

As a marginal finding, we report the entomopathogenic fungus *B. bassiana* from the gut of *S.* cf. *laevigatus*. This fungus is used as biological pest-control agent against several species of phytoparasitic mites (Jiang et al. 2019). However, the lack of evident disease symptoms and the presence of *B. bassiana* in the gut contents of the herein examined individuals indicates that hyphae of this fungus may serve as a food source for *S.* cf. *laevigatus*, implying some degree of tolerance to toxic molecules, such as beauvericin and beauverolid (Pedrini 2022) and the active role of mites in fungal dispersion. This agrees with former observations reporting the asymptomatic association of *B. bassiana* with *Paradamaeus clavipes* (Renker et al. 2005).

The occurrence of yeasts in the gut of oribatid mites is not rare, although these microfungi are scarcely reported from soil (Princz et al. 2010; Molva et al. 2019). Our results suggest that *Z.* cf. *floridana* and *S.* cf. *laevigatus* were feeding on *S. cerevisiae* in soil, as it has been previously observed in various feeding preferences tests (van Bronswijk 1971; Andersen 1991; Princz et al. 2010; Molva et al. 2019). Moreover, its occurrence as an endosymbiotic fungus is plausible since several yeast species have been reported as beneficial to mites establishing mutualistic relationships, that provide the host with vitamins or sterols (Ganter 2006; Douglas 2009; Chandler et al. 2012).

The presence of *Filobasidium* sp., *Roussoella* sp. and Sclerotiniaceae sp. in the gut content of *S.* cf. *laevigatus* resembles previous work describing the high abundance of these fungi in the soil and in association with plants, particularly in the cases of saprophagous *Roussoella* sp. (Liu et al. 2014) or the putative facultative phytopathogens Sclerotiniaceae sp. (Adams and Ayers 1979). However, the chemical interactions between host plants, phytopathogens, and mites (e.g., *S.* cf. *laevigatus*) are still open for research.

In contrast to our hypothesis, our observations indicate that *Z.* cf. *floridana* and *S.* cf. *laevigatus* have similar fungal-based dietary inclinations, as both oribatid mites share preference for *Cladosporium* spp. and *Mortierella* spp. Nevertheless, we observed distinct degrees of preference between both oribatid taxa, which may facilitate an intensive appropriation of fungal resources based on resource partitioning. This feeding behavior might represent a suitable way to avoid competition in a stressful environment such as arid soils. Finally, our findings confirmed that the molecular approach is very useful for the establishment of in situ (in the environment) feeding preferences. However, it is necessary to conduct complementary studies, including a deeper microbiome analysis to discard food sources from endosymbionts. This is needed for understanding how oribatid mites affect fungal-based food-webs in arid and semiarid lands. Microbiome plays an important role in digesting preferred fungal prey due to the molecular specificities of the fungal cell wall (Smrž et al. 2016), it is likely that *Z.* cf. *floridana* and *S.* cf. *laevigatus* share mutualistic microbiota capable of degrading similar hyphae components.

## Supplementary Information

Below is the link to the electronic supplementary material.

Supplementary file1 (DOCX 21 KB)
