# Supplementary material for: Fungal feeding preferences and molecular gut content analysis of two abundant oribatid mite species (Acari: Oribatida) under the canopy of Prosopis laevigata (Fabaceae) in a semi-arid land
Source: Exp Appl Acarol. 2023 Apr 18;89(3-4):417–32. doi: 10.1007/s10493-023-00790-7 (PMC10167177; doi:10.1007/s10493-023-00790-7)
Supplement: Supplementary file 1 — Supplementary file1 (DOCX 21 KB) [file 10493_2023_790_MOESM1_ESM.docx]

|  |  | **Conserved terrace** | | | | | |
| --- | --- | --- | --- | --- | --- | --- | --- |
| **Genera** | **Codes** | **1** | **2** | **3** | **4** | **5** | **6** |
| *Aphelacarus* sp. | Aphel | 5 |  | 8 | 3 | 6 |  |
| *Brachychthonius* sp. | Brach | 2 | 1 | 2 |  | 1 | 2 |
| *Cosmochthonius* sp. | Cosmo | 2 |  | 1 | 1 | 1 |  |
| *Perlohmannidae* sp. | Perlo |  |  | 1 | 1 |  |  |
| *Rhysotritia* sp. | Rhyso | 3 |  |  |  | 1 | 3 |
| *Plesiodamaeus* sp. | Plesio | 1 |  | 2 |  |  |  |
| *Epidamaeus* sp. | Epida |  | 3 | 5 |  |  | 1 |
| *Oppia* sp. | Opp | 5 |  | 7 | 2 |  | 5 |
| *Tectocepheus* sp. | Tecto |  |  | 1 |  |  | 2 |
| *Scapheremaeus* sp. | Scaph |  |  |  | 1 |  |  |
| *Eremaeozetes* sp. | Erema | 3 | 2 |  | 3 | 5 |  |
| *Scutovertex* sp. | Scuto |  | 1 | 2 |  | 1 |  |
| *Zygoribatula* sp. | Zygor | 15 | 11 | 20 | 7 | 8 | 14 |
| *Parakalumnidae* sp. | Parak | 2 |  | 1 |  |  | 1 |
| *Scheloribates* sp. | Schel | 10 | 12 | 8 | 10 | 13 | 8 |
| *Ceratozetes* sp. | Cerat |  | 1 |  |  | 1 |  |
| *Galumna* sp. | Galum | 3 | 3 |  | 2 | 5 | 2 |
| **Total** |  | 51 | 34 | 58 | 30 | 42 | 38 |

Supplementary table S1. Abundance of the different oribatid mite taxa found under the canopy of *Prosopis laevigata* (N=6) in both terraces (conserved terrace and degraded terrace)

|  |  | **Degraded terrace** | | | | | |
| --- | --- | --- | --- | --- | --- | --- | --- |
| **Genera** | **Codes** | **1** | **2** | **3** | **4** | **5** | **6** |
| *Aphelacarus* sp. | Aphel |  | 2 | 1 | 1 |  | 2 |
| *Brachychthonius* sp. | Brach | 3 |  |  | 1 |  | 1 |
| *Cosmochthonius* sp. | Cosmo | 1 |  | 1 |  |  | 2 |
| *Perlohmannidae* sp. | Perlo |  |  |  |  |  |  |
| *Rhysotritia* sp. | Rhyso |  |  | 1 |  |  |  |
| *Plesiodamaeus* sp. | Plesio |  | 1 |  |  |  | 1 |
| *Epidamaeus* sp. | Epida |  |  |  | 2 |  |  |
| *Oppia* sp. | Opp |  |  |  | 3 | 1 |  |
| *Tectocepheus* sp. | Tecto |  |  | 2 |  | 1 |  |
| *Scapheremaeus* sp. | Scaph |  |  | 1 |  |  |  |
| *Eremaeozetes* sp. | Erema |  | 2 |  |  | 2 | 1 |
| *Scutovertex* sp. | Scuto |  |  | 3 | 1 |  |  |
| *Zygoribatula* sp. | Zygor |  | 5 | 4 | 3 | 4 | 6 |
| *Parakalumnidae* sp. | Parak |  | 1 |  |  |  |  |
| *Scheloribates* sp. | Schel | 3 |  | 1 | 5 | 2 | 3 |
| *Ceratozetes* sp. | Cerat | 1 |  | 1 |  | 2 |  |
| *Galumna* sp. | Galum | 1 |  |  | 2 |  |  |
| **Total** |  | 9 | 11 | 15 | 18 | 12 | 16 |
